# Experimental Tracheal Replacement Using 3-dimensional Bioprinted Artificial Trachea with Autologous Epithelial Cells and Chondrocytes

**DOI:** 10.1038/s41598-019-38565-z

**Published:** 2019-02-14

**Authors:** Jae-Hyun Park, Jeong-Kee Yoon, Jung Bok Lee, Young Min Shin, Kang-Woog Lee, Sang-Woo Bae, JunHee Lee, JunJie Yu, Cho-Rok Jung, Young-Nam Youn, Hwi-Yool Kim, Dae-Hyun Kim

**Affiliations:** 1 Department of Veterinary Surgery, College of Veterinary Medicine, Konkuk University, 120 Neungdong-ro, Gwangjin-gu, Seoul 05029 Republic of Korea; 2 Division of Cardiovascular Surgery, Severance Cardiovascular Hospital, Yonsei University College of Medicine, 50-1 Yonsei-ro, Sedaemun-gu, Seoul 03722 Republic of Korea; 3 Severance Biomedical Science Institute, Yonsei University College of Medicine, 50-1 Yonsei-ro, Sedaemun-gu, Seoul 03722 Republic of Korea; 4 Department of Nature-Inspired Nanoconvergence System, Korea Institute of Machinery and Materials, 156 Gajeongbuk-Ro, Yuseong-Gu, Daejeon 34103 Republic of Korea; 5 Department of Biomedical Engineering, School of Integrative Engineering, Chung-Ang University, 84 Heukseok-Ro, Dongjak-Gu, Seoul 06974 Republic of Korea; 6 Gene Therapy Research Unit, Korea Research Institute of Bioscience and Biotechnology, 125 Gwahak-ro, Yuseong-gu, Daejeon Republic of Korea

## Abstract

Various treatment methods for tracheal defects have been attempted, such as artificial implants, allografts, autogenous grafts, and tissue engineering; however, no perfect method has been established. We attempted to create an effective artificial trachea via a tissue engineering method using 3D bio-printing. A multi-layered scaffold was fabricated using a 3D printer. Polycaprolactone (PCL) and hydrogel were used with nasal epithelial and auricular cartilage cells in the printing process. An artificial trachea was transplanted into 15 rabbits and a PCL scaffold without the addition of cells was transplanted into 6 rabbits (controls). All animals were followed up with radiography, CT, and endoscopy at 3, 6, and 12 months. In the control group, 3 out of 6 rabbits died from respiratory symptoms. Surviving rabbits in control group had narrowed tracheas due to the formation of granulation tissue and absence of epithelium regeneration. In the experimental group, 13 of 15 animals survived, and the histologic examination confirmed the regeneration of epithelial cells. Neonatal cartilage was also confirmed at 6 and 12 months. Our artificial trachea was effective in the regeneration of respiratory epithelium, but not in cartilage regeneration. Additional studies are needed to promote cartilage regeneration and improve implant stability.

## Introduction

The most commonly occurring tumours of the trachea are known as adenoid cystic carcinomas, squamous cell carcinomas, and so on. When a malignant tumour causes narrowing of the trachea, the damaged area is excised and end-to-end anastomosis is performed; however, this can only be applied to lesions 6 cm or less in size^1^. Although lesions larger than 6 cm are now treated with a stent, there is a risk of migration, corrosion, or haemorrhage, and stents are difficult to use permanently. Various methods, have been tried to overcome these drawbacks so that stents can be successfully applied to lesions larger than 6 cm^2^.

Various methods, mainly using tissue grafting or tissue engineering, have been attempted. Early attempts used artificial implants, but attempts to produce effective and stable implants failed^3–5^. Then, researchers attempted to use a heterologous tissue, which resulted in long-term immunosuppression^6^. A method using an autologous tissue, such as aortic or oesophageal tissue, was also tried^7^, but these stents did not have the strength of the tracheal cartilage, and could not withstand the pressure during breathing. In addition, the surrounding tissue may unite with the stent and cause stenosis or haemorrhage^8^.

In the present days, artificial trachea has been studied to overcome such problems. To fabricate an artificial trachea for tracheal regeneration, the goal is to produce flexibility and strength resembling normal tracheas, and to produce a characteristic ciliated epithelium. For decades, various methods have been used to make artificial tracheas suitable for these conditions^9,10^. One method involved fabricating and mounting of cylindrical implants^9,11^. Since these implants cannot be combined with surrounding tissues, infection, dislodgement, immune reaction, migration, obstruction and other problems have occurred and epithelialisation has not progressed^12^. The most actively studied method for fabricating artificial trachea is tissue engineering, and biodegradable synthetic polymers are used to make tubular scaffolds. A scaffold made using tissue engineering tends to have a smooth vascularization and less obstruction when compared to foreign materials, allografts, and autogenous grafts. Currently, studies are underway in various directions, including those that regenerate tracheal epithelium^13^.

In recent years, due to the development of 3-dimensional (3D) printing technology, various attempts have been made to use this technology in tissue engineering^14^. Biodegradable materials, such as polycaprolactone (PCL), polyglycolic acid (PGA), polylactic acid (PLA), and poly(lactic-co-glycolic) acid (PLGA), used in 3D printing have strengths similar to the tracheal cartilage; therefore, various attempts are being made to apply 3D printing technology to artificial trachea research^15^. In addition, given the development of bio-printing technology, living cells can be added to hydrogel for printing, and cells, such as chondrocytes or stem cells, can be printed together in the production of artificial tracheas^16^.

Through this study, we tried to construct artificial tracheas similar to the original tracheal structure using 3D printing and observe them for any unusual characteristics at 3, 6, and 12 months. This study is the first to treat tracheal lesions using 3D printed artificial trachea with autologous epithelial cells and chondrocytes, and there have been few studies examining long-term effects of artificial tissue over one year. Respiratory epithelia and cartilage chondrocytes, which play the most important role in the structure of the trachea, were cultured to produce hydrogel for bio-printing. An artificial trachea was produced using 3D printing with a biodegradable polymer. In addition, an artificial trachea was implanted in a well-known trachea scaffold partial resection model^17^, and respiration scoring, x-ray, computed tomography (CT), endoscopy, and histological examination were performed.

## Results

### Bioprintability of artificial trachea

**Figure 1:**
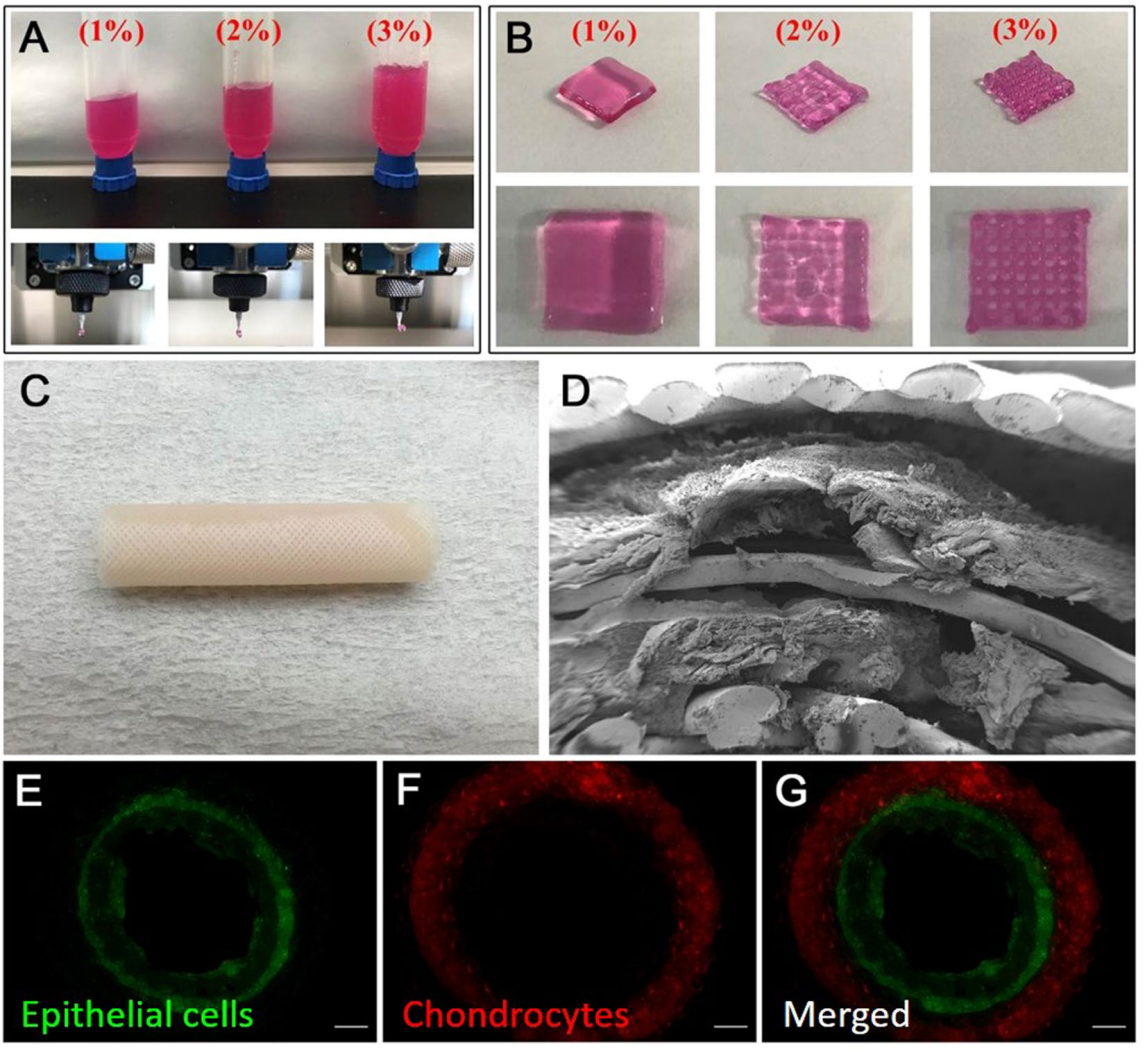
3D printed alginate hydrogel and artificial trachea. (**A**) Alginate hydrogel being extruded at 300 um nozzle. (**B**) Optical image of 3D alginate cube type (16 × 16 × 2 mm^3^). The higher concentration of alginate hydrogel providing more precise and porous cube type. (**C**) Gross image of the artificial trachea fabricated using a 3D bio-printer. (**D**) Scanning electron microscopic image. From the bottom: first, porous PCL layer; second, epithelial cell layer; third, non-porous PCL layer; fourth, chondrocyte layer; and fifth, porous PCL layer are clearly seen. (**E–G**) Fluorescent microscopic images using green dye for the epithelial cells (**E**) and red dye for chondrocytes (**F**); and merged image (**G**) reveals that the 2 hydrogel layers are completely separated.

Grid patterned alginate hydrogels with a concentration of 1, 2, and 3% (w/v) was printed through a 300 µm nozzle (Fig. 1A). In the case of hydrogel at 1% and 2% of alginate solution, the grid pattern was not well formed: the strand was deconstructed and they are not suitable for further studies (Fig. 1B). Accordingly, we could obtain the desired grid pattern cube at a concentration of 3% which maintains its shape after printing (Fig. 1B). Meanwhile, cell viability was significantly reduced at concentrations above 3%, thereby excluded from the evaluation (data not shown).

The 3D bio-printed artificial graft turned out just as we had planned (Fig. 1C). The SEM images revealed that the hydrogel containing epithelial cells on the inner layer and the hydrogel containing chondrocytes on the outer layer were printed on the boundary of the PCL layer (Fig. 1D). Fluorescence microscopy of scaffolds stained with Cell Tracker™ resulted in a complete separation of the green-stained epithelial cells and red-stained chondrocytes layers (Fig. 1E–G).

**Figure 2:**
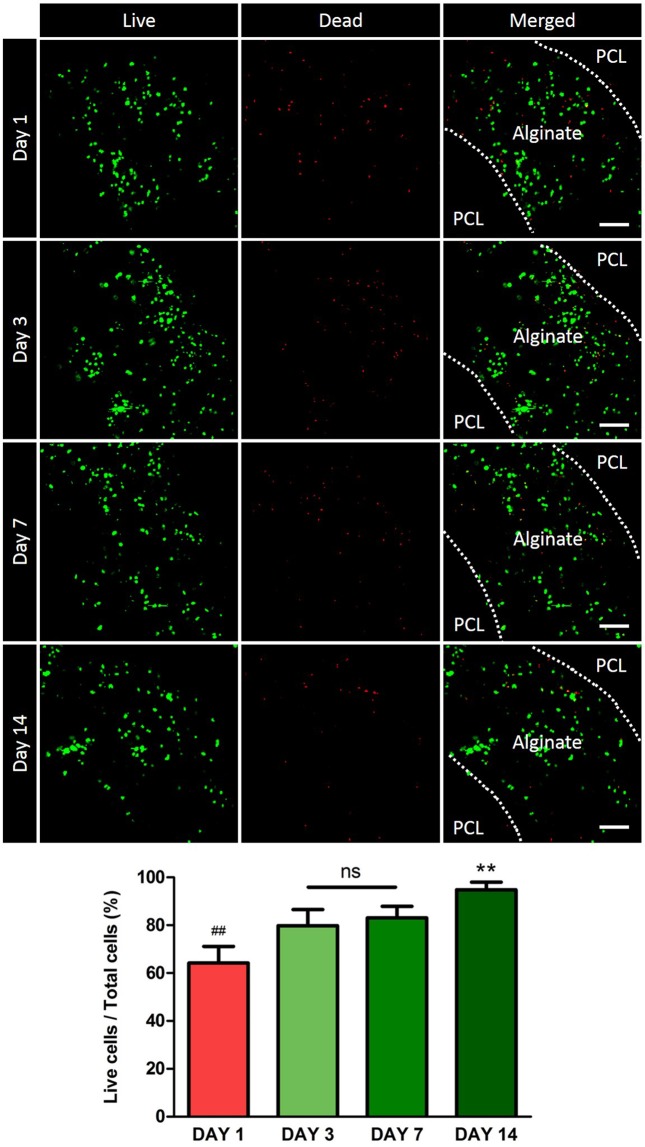
*In vitro* Live/Dead Assay. The live cells and the dead cells are green and red, respectively. From the 3^rd^ day of culture, the ratio of live cells is maintained at 80% or more. On day 14, alginate hydrogel is degraded and cells are observed at the PCL scaffold. Scale bars indicate 100 μm.

In the first Live/Dead Assay, the number of live cells increased overtime. The ratio of dead cells on day 1 was about 30%, but from day 3, it was maintained below 20%, which confirmed the absence of problem with regard to survival of cells in the hydrogel (Fig. 2).

### Clinical evaluation of the artificial trachea

A summary of the results is shown in Table 1. No animals died during the surgical procedure, and all awakened from the anaesthesia. The survival ratio of the control animals in the first 3 months was three of six animals. Meanwhile, in experimental group, the survival ratio in the first 3 months was five of six, and one more rabbit died within the next 3 months. The rabbits that died showed symptoms of anorexia and diarrhoea. The other animals were euthanized on a planned date. There were no deaths in the 12-month observation group. The surviving rabbits had no specific clinical signs, and euthanasia was performed in time.

**Table 1:** Clinical and Pathological Results of Animals with Tracheal Replacement using 3-D Printed Artificial Tracheas.

Group, animal no.	State	Follow-up	Complication	Stenosis rate (%)	Respiratory scoring	Epithelialisation	Cartilage regeneration
**Control**							
1	Sacrificed	45 days	Diarrhea, anorexia	39.757	3		
2	Died	3 months		29.712	1		
3	Sacrificed	44 days	Nasal discharge, anorexia	76.428	3		
4	Sacrificed	3 months		38.271	2		
5	Died	3 months		22.470	2		
6		34 days	Nasal discharge, anorexia	70.471	3		
**Experimental**							
7	Sacrificed	3 months		13.529	0	Mucociliary	
8	Sacrificed	3 months		23.357	1	Squamous metaplasia	
9	Sacrificed	3 months		9.477	0	Mucociliary	
10	Died	13 days	Diarrhoea, anorexia		1		
11	Sacrificed	3 months		25.269	0	Mucociliary	
12	Sacrificed	3 months		27.547	1	Mucociliary	
13	Sacrificed	6 months		22.811	0	Mucociliary	Islet
14	Sacrificed	6 months		−26.681	0	Mucociliary	Islet
15	Sacrificed	6 months		14.637	1	Mucociliary	Islet
16	Sacrificed	6 months		14.799	1	Mucociliary	Islet
17	Died	21 days	Diarrhoea, anorexia		1		
18	Sacrificed	6 months		7.423	1	Mucociliary	Islet
19	Sacrificed	1 years		7.542	0	Mucociliary	Islet
20	Sacrificed	1 years		5.517	0	Mucociliary	Islet
21	Sacrificed	1 years		7.112	0	Mucociliary	Islet

Immediately after surgery, all rabbits showed continuous crackles or stridor. At the time of euthanasia, the mean respiration score was 2.20 ± 0.84 points in the control group; most rabbits showed intermittent crackles or stridor in the resting state, and laboured respiration was observed in some rabbits. On the other hand, in the experimental group, the average respiration score was 0.47 ± 0.52 points, and normal respiration was observed in most animals in the experimental group. Only a few rabbits in the experimental group showed intermittent crackles or stridor when excited.

Radiographs taken immediately after surgery showed an increase in opacity at implant sites in all rabbits. In the control group, the decreasing diameter ratio was 46.19 ± 22.10% on average, which was larger than the 11.72 ± 13.81% seen in the experimental group. In particular, the rate of decrease in tracheal diameter in the 12-month observation group was 6.72 ± 1.07%, which was close to the diameter of a normal trachea.

**Figure 3:**
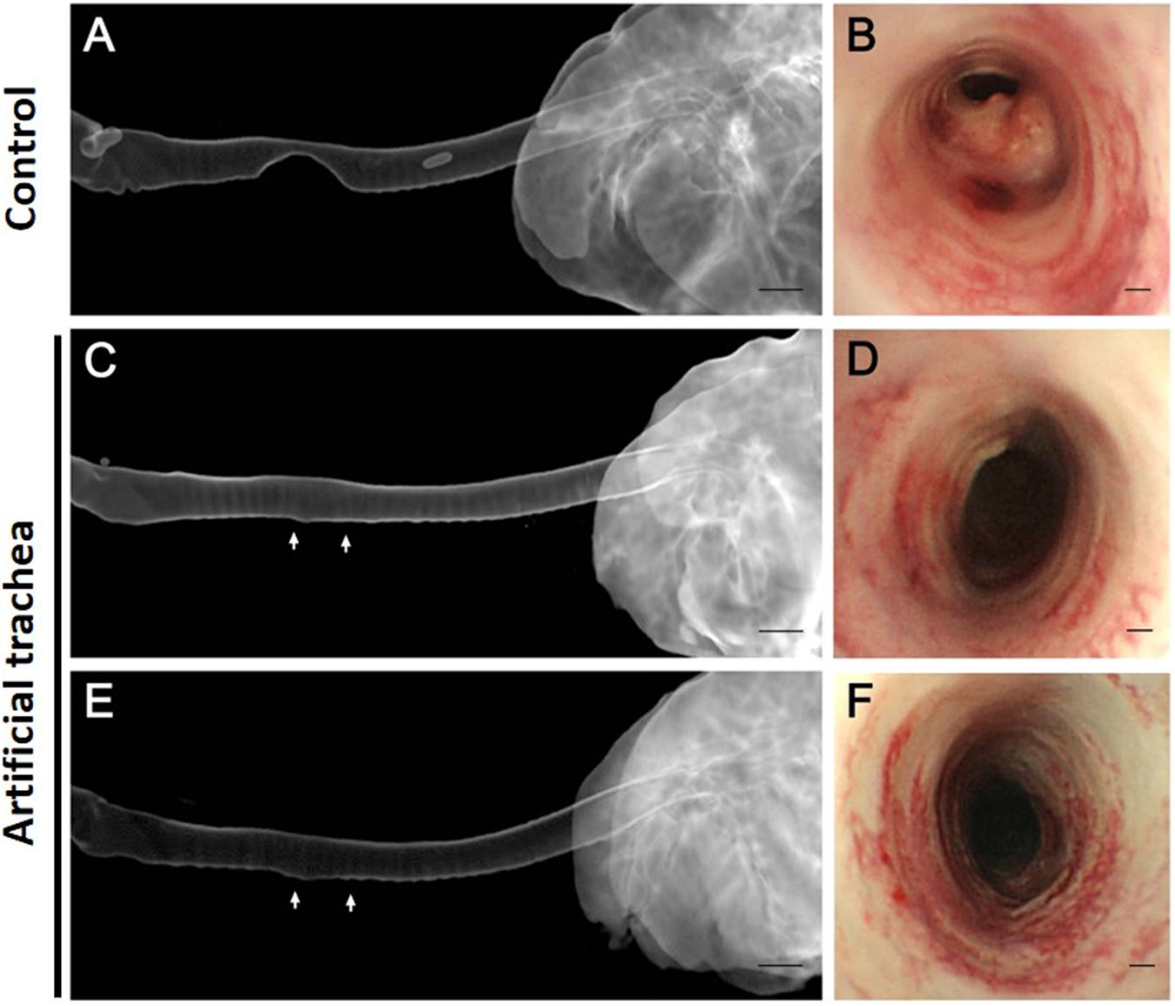
Computed tomography and bronchoscopic images. (**A**) Control group (animal no. 2), (**B**) Control group (animal no. 4), (**C**) experimental group (animal no. 13), (**D**) experimental group (animal no. 7), (**E**) experimental group (animal no. 19) and (**F**) experimental group (animal no. 20). On CT images, the tracheal diameter of the control group (**A**) is significantly reduced. On the other hand, the tracheal diameter of the experimental group maintains airway patency after 6 (**C**) and 12 months (**E**). On bronchoscopic images, the lumen of the trachea is narrowed and massive granulation tissue proliferation is observed in the control group (**B**). However, the tracheal lumen in the experimental group observed at 3 (**D**) and 12 months (**F**) is not narrowed, and the mucosa of the trachea is similar to the normal trachea. The grafted area was pointed with white arrows. Scale bars indicate 10 mm (**A,C,E**), and 500 μm (**B,D,F**), respectively.

On CT images, we observed that the inner diameter of the graft site was significantly reduced in the control group (Fig. 3A). On the other hand, the tracheal inner diameters in the 6-month survival group and 12-month survival group did not decrease (Fig. 3C,E).

On bronchoscopic images, the diameter of the trachea was markedly narrowed, and the granulomatous tissue was proliferating to block some of the trachea in the control group (Fig. 3B). However, the inner diameter showed almost no decrease, and internal structures very similar to a normal trachea were seen in the experimental group (Fig. 3D,F).

### Histological regeneration of the artificial trachea

**Figure 4:**
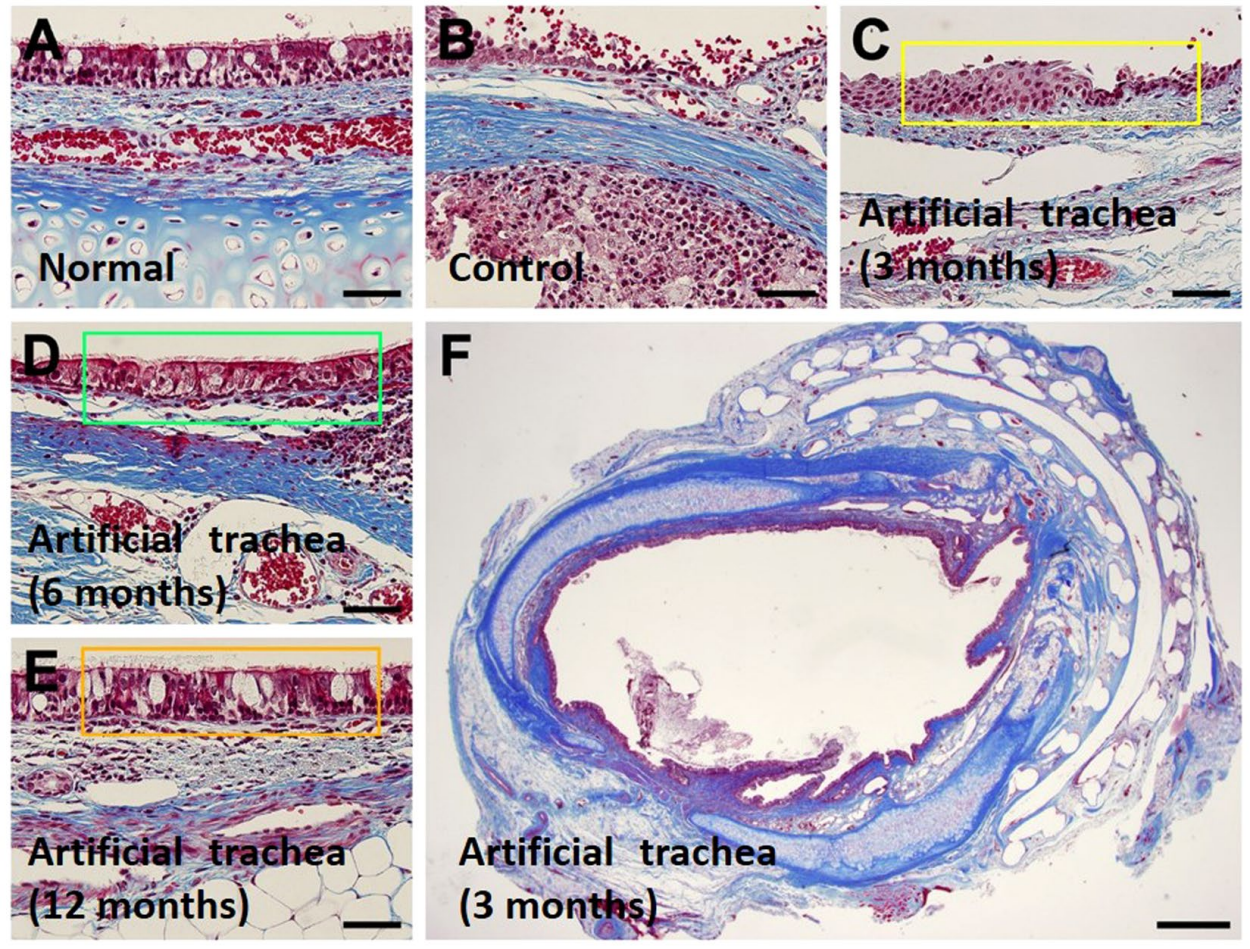
Histopathologic images of epithelial regeneration. Compared with the normal tracheal epithelium (**A**), the control group (animal no. 5) does not show epithelial regeneration (**B**). However, the experimental group shows epithelial regeneration at 3 months, and 1 animal shows incomplete epithelial regeneration with squamous metaplasia (**C**, animal no. 8). The animals at 6 (**D**, animal no. 14) and 12 months (**E**, animal no. 21) show complete epithelial cell regeneration. (**F**) A whole trans-sectional image at 3 months in the experimental group (animal no. 9) also shows complete epithelial regeneration. (Masson’s trichome staining, the bars in subpanels A-E indicate 50 µm and that in subpanel F indicates 1 mm) The epithelial regeneration was pointed with green and yellow boxes in (**D,E**).

There was no regeneration of epithelial cells in the staining of the control group and an inflammatory reaction was observed, which resulted in narrowing of the lumen of the trachea. On the other hand, in the experimental group, ciliated epithelial cells were observed in all the rabbits over 3 months, covering the inside of the scaffold, and forming a similar structure to a normal trachea (Fig. 4).

**Figure 5:**
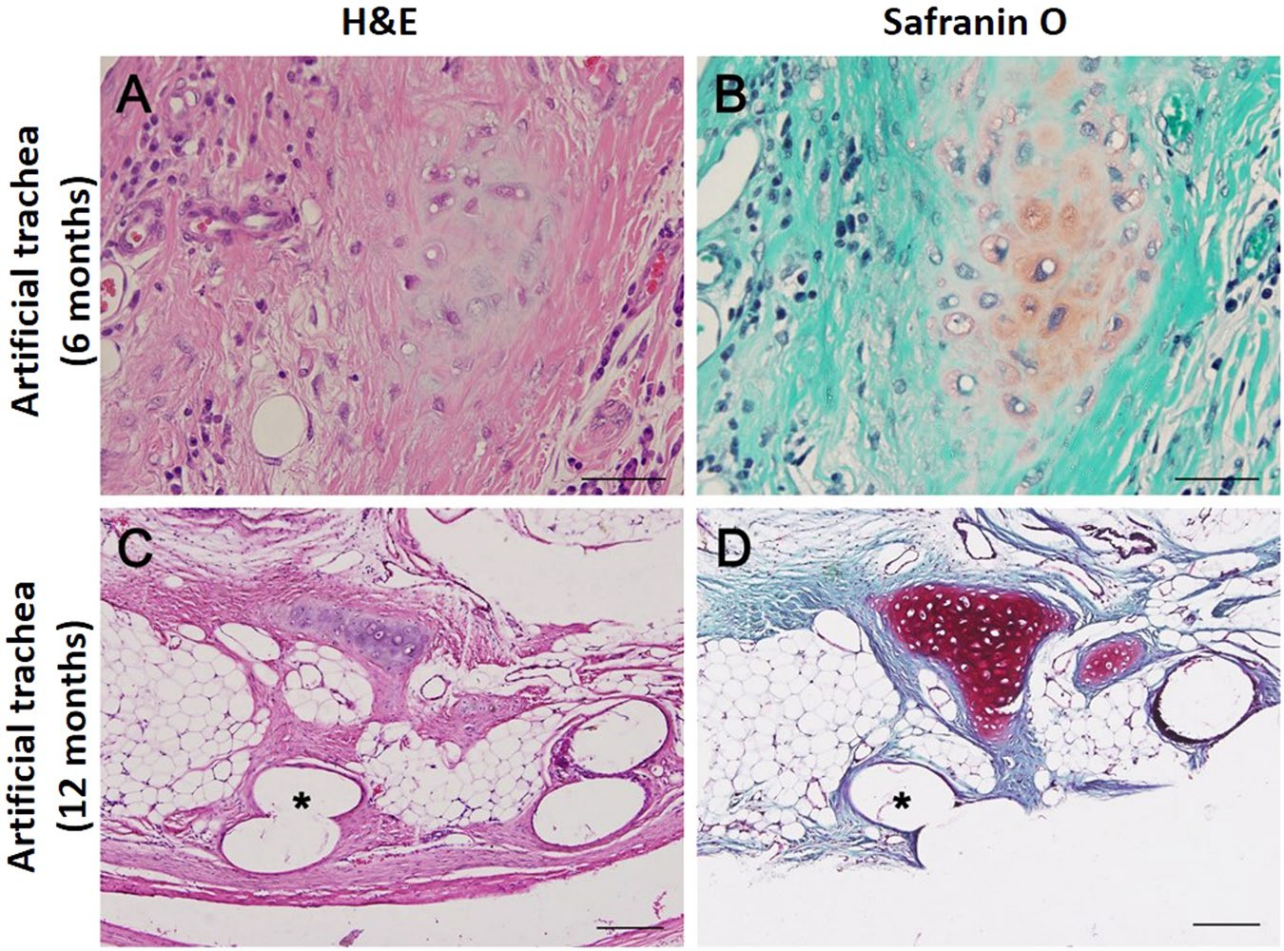
Histopathologic images of tracheal cartilage regeneration. The formation of immature cartilaginous islets is observed at 6 (**A** and **B**, animal no. 15) and 12 months (**C** and **D**, animal no. 20). (A and C = haematoxylin and eosin staining, B and D = safranin O staining, the asterisk indicates PCL strand of fifth layer, all bars indicate 50 µm).

Cartilaginous tissue was not observed in the experimental group at 3 months. However, immature cartilage islets were observed in the experimental group at 6 and 12 months (Fig. 5). Formation of c-shaped cartilage was not observed.

## Discussion

In the case of the trachea, the most important structures for performing the role are the cartilage and epithelium. The cartilage should be strong enough to withstand the pressure during breathing and be flexible^2^. The epithelium is composed of characteristic ciliated epithelial cells and can be seen from the inside of the trachea and nasal cavity. For tracheal transplantation, allograft and autograft have been conventionally used.

In allografts, the methods of chemically treating, lyophilizing, or freezing the trachea^18^ and other tissues like the aorta are used^19^. Experiments have also been carried out on allografts of tracheas treated in various ways^20^. However, in the case of an allograft, the vascularization does not progress^21^, and eventually stenosis due to fibrosis occurs^22^. There are also problems encountered by patients with immune reactions^23^. Autogenous grafts have also been actively studied. Initially, autogenous grafts were combined with foreign material implants^9,24^. In some of these experiments, epithelialization and cartilage formation were observed, but stenosis eventually occurred. Autogenous tissue has also been transplanted into a patch^25^. However, in non-congenital stenosis, fibrosis and contraction proceeded, and obstruction of the trachea occurred.

In this experiment, to overcome such problems of allograft and autograft, we used PCL as a biodegradable synthetic polymer for tissue engineering. PGA is a conventionally used polymer because of its high porosity, which can induce cell infiltration and neovascularization and can be absorbed at a relatively accurate time^26^. However, due to its short absorption time, it is difficult to use for long-term therapeutic effect, and it also has low strength^26^. On the other hand, PCL has a low porosity, but its long absorption time and strength are superior to PGA, so it has long-term applicability; some studies have also shown that low porosity promotes chondrogenesis^27^. A total of 3 PCL layers were applied in this experiment. The PCL of the innermost and outermost layers was fabricated with a grid pattern with pores to promote cell filtration and neovascularisation. The middle layer was made into a cylindrical shape with no pores, so that the epithelial cells and cartilage layer could be separated and physical strength could be increased. The elastic modulus of PCL can be easily modulated by controlling the molecular weight. The PCL construct which we used in this study was designed to have similar mechanical properties to that of rabbit trachea. Thus, to apply our system to larger animal models or humans, we should control the elastic modulus of the artificial trachea, by using PCL with different molecular weight (i.e. pig tracheal cartilage = 1.74 ± 0.85 MPa^28^, human tracheal cartilage = 16.92 ± 8.76 MPa^29^).

**Figure 6:**
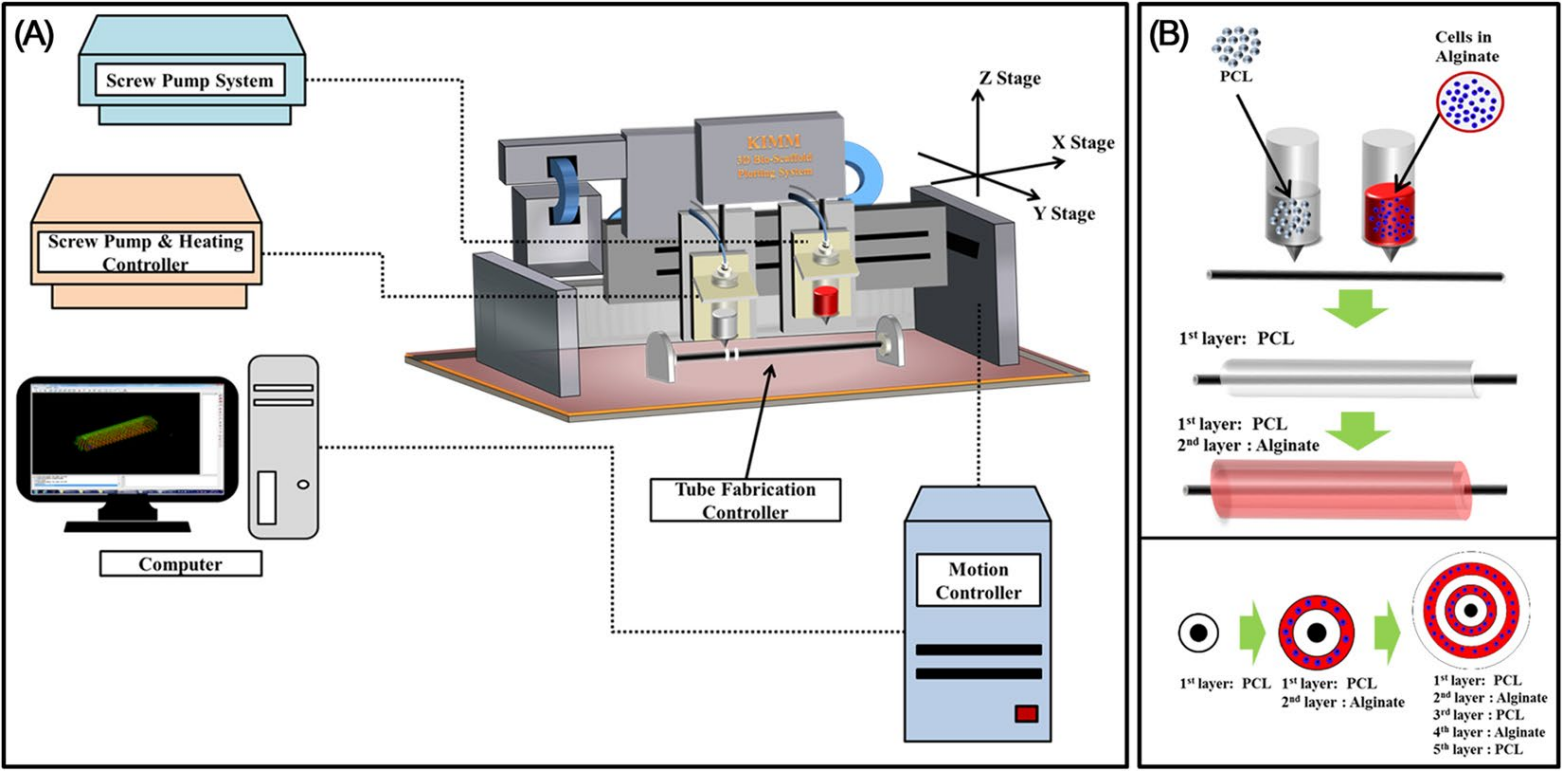
System components and bioprinting process. (**A**) Schematic graph of 3D bio-scaffold plotting system. (**B**) Detailed fabrication process of artificial trachea using bio plotting system. First, porous PCL layer; second, cell-laden alginate; third, non-porous PCL layer; fourth, cell-laden alginate; and fifth, porous PCL printing on tube type module.

Between each PCL layers, two different types of autogenous cells were placed with alginate hydrogel. In this study, we choose alginate hydrogel for cell printing, as several groups have revealed about the biocompatibility/biodegradability of alginate^30^, and used for tracheal regeneration^31^. Optimization of the concentration of alginate is important as minimum shape-stability is necessary in order to maintain the cylindrical hydrogel shape and to prevent from flowing down (Fig. 6B). Also, high alginate concentration has harmful effect on cell viability. We previously measured the rheological properties of alginate + CaCl_2_ hydrogel^32^, and we used a rheologically optimized hydrogel. We confirmed the majority of the cells were viable after gelation (Fig. 2). Also, we have confirmed that our 3D-printing process itself has no effect on cell viability, by performing the live and dead assay at day 1 on cells in film typed alginate + CaCl_2_ hydrogel without printing process (Supplementary Fig. S1).

A living tissue is composed of at least two types of cells. In other words, it is recommended that any artificial graft must contain two or more kinds of cells in order to perform its function *in vivo*. Trachea is also a complex tissue composed of epithelium, cartilage and also a membranous structure with smooth muscle cells in the dorsal side. In this study, we focused on epithelium and cartilage regeneration, thus nasal epithelial cells in the inside layer, and auricular cartilage cells in the outside layer was applied. However, applying two or more types of cells to implants has been a challenge^33^. In this regard, recent bio-printing techniques are considered a very effective solution. When 3D printing is performed using hydrogel-cultured cells, various kinds of cells can be applied to the scaffold^33^. Furthermore, it has been confirmed through the Live/Dead Assay that there was no problem with cell survival in hydrogel^34^.

To evaluate the *in vivo* therapeutic efficacy of our artificial trachea, rabbit partial resection model was used, as it is a commonly-used animal model for airway transplantation research^35,36^. Rabbit partial resection model is an economical and commonly used animal model compared to pig or sheep. Also, the method of harvesting rabbit-derived nasal epithelial cells and auricular chondrocytes is well-established. Refer to previous studies, spontaneous regeneration is limited if the airway is dissected more than 50%^36^. In particular, nasal epithelial cell cultures were used to transplant trachea-like ciliated epithelial cells without damaging the tracheas of the rabbit before surgery, and epithelial cells were successfully formed in all the experimental groups. We believe that this is the effect of transplantation of epithelial cells into scaffolds and that there is a direct correlation with early survival rate. Cartilage also plays an important role for a successful transplantation. In this experiment, the auricular cartilage cell was cultured and applied to the scaffold, but no cartilage formation was observed in the 3-month observation group, and some neonatal cartilage could be observed at 6 and 12 months. Other studies using rabbit ear chondrocytes for tracheal grafts also revealed a cartilaginous island similar to that of the native trachea at 6 months, but it did not produce a complete c-shape^36^. Thus, more effective methods are needed to reduce the time it takes for the cartilage to regenerate. For example, stem cells and the extracellular matrix are involved in cartilage regeneration in the tracheas^37^, so further studies utilizing mesenchymal stem cells or decellularized cartilage powder^38^ are needed in the future. We did not focus on this study, but the regeneration of connective tissue is also an important point of tracheal repair, thereby further studies would be necessary.

## Conclusions

In this experiment, an artificial trachea was constructed using a 3D bio-printing technique and was successfully transplanted with two different types of autogenously isolated cells, epithelial cells and chondrocytes. The artificial trachea was successfully engrafted into the partially resected trachea, resulting epithelialization and formation of cartilage islet. This resulted thirteen of fifteen animals’ survival until 12 months without specific respiratory signs in the experimental group, while the control group showed only four of six animals’ survival. Our research could inform that our 3D-printed artificial graft containing autogenous cells is stable enough for a long time about a year, and could provide a platform to apply with several different types of cells or suitable biomaterials to treat tracheal diseases.

## Materials and Methods

This animal study was approved by the Institutional Animal Care and Use Committee of Yonsei University Health System (publication no. 2015-0361, 2015). This study was performed according to the ARRIVE guidelines and the National Institutes of Health Guide for the Care and Use of Laboratory Animals.

### Preparation of autologous nasal epithelial cells and auricular chondrocytes

For primary culture of rabbit nasal epithelial cells, 15 mature male New Zealand White rabbits (3.0–3.5 kg) were injected with 5 mg/kg xylazine and 10 mg/kg Zoletil^®^ intramuscularly at 15 min intervals for anaesthesia. Nasal mucosa was harvested from the nasal cavity using a $\varnothing$ = 4 mm skin biopsy punch and then stored in phosphate-buffered saline (PBS) containing 1% penicillin-streptomycin (Thermo Fisher Scientific, MA, USA) for 30 min. Submucosal tissue was eliminated on a sterilised petri dish on a clean bench. The harvested tissue was incubated with 0.2% (w/v) collagenase type II (Thermo Fisher Scientific, MA, USA) in Ham’s F-12 medium (Welgene, Daegu, Korea) for 24 h. It was then filtered through a 100 µm nylon cell strainer (BD Biosciences, NJ, USA) and centrifuged at 1500 rpm for 5 min. Subsequently, the supernatant was discarded. Nasal epithelial cells were seeded in a 100∅ cell culture dish (SPL Life Sciences, Gyeonggi-do, Korea) with Ham’s F-12 medium supplemented with 10% fetal bovine serum (FBS) (GE Healthcare, UT, USA), 1% penicillin-streptomycin, 10 µg/ml amphotericin B (Enzo Life Sciences, NY, USA), 50 µg/ml gentamicin (Daesung Microbiological Labs, Gyeonggi-do, Korea), 0.5 µg/ml hydrocortisol (Sigma-Aldrich, MO, USA), 5 ng/ml epidermal growth factor (EGF) (ProSpec, NJ, USA), 1.5 µg/ml bovine serum albumin (BSA), (MP Biomedicals, CA, USA), and 1 × insuline transfferin selenium(ITS) + 3 solution (Sigma-Aldrich, MO, USA).

For the primary culture of rabbit auricular cartilage cells, the ear of the rabbit was cut approximately 2 × 2 cm in size using Metzenbaum scissors and stored in PBS containing 1% penicillin-streptomycin for 30 min. Perichondrium was eliminated as much as possible in a sterilised petri dish on a clean bench. Harvested auricular cartilage was incubated with 0.2% (w/v) collagenase type II in Ham’s F-12 medium for 24 h. It was then filtered through a 100 µm nylon cell strainer (BD Biosciences, NJ, USA) and centrifuged at 1500 rpm for 5 min. Subsequently, the supernatant was discarded. The auricular cartilage cells were seeded in a 100∅ cell culture dish (SPL Life Sciences, Gyeonggi-do, Korea) with Dulbecco’s Minimal Essential Medium with high glucose (Welgene, Daegu, Korea) supplemented with 10% FBS, 1% penicillin-streptomycin, 10 µg/ml amphotericin B, 50 µg/ml gentamicin, and 25 µg/ml L-ascorbic acid(Sigma-Aldrich, MO, USA). Passage 2 cells were used for manufacturing the artificial trachea.

### Bioprinting process

The 3D printing was performed using a 3D bioprinter (KIMM &Protek Korea, Daejeon, Korea).The bioprinter consists of a ‘screw pump system’ for hydrogel printing, a ‘screw pump and heating controller’ for PCL printing, a ‘tube fabrication controller’ for tube shape formation, and a ‘motion controller’ that receives information about the design from the main computer to control the nozzles to construct the desired structure (Fig. 6).

The 3D printability of the hydrogel was optimized using three different concentrations (1%, 2%, and 3% w/v) of sodium alginate hydrogels, by stacking 300 μm grid shapes to constructthe 16 × 16 × 2 mm cubes.For the fabrication of the scaffold, PCL (Sigma-Aldrich, MO, USA, Catalog no.440744) polymer pellets were melted at 100–130 °C in a heating cylinder and ejected through a heated nozzle. The thickness of PCL strands was 2.5 mm. To make a hydrogel for printing, after counting 1 × 10^7^cells, they were suspended in serum free media and made a total of 8.2 ml of hydrogel. Then, 1.5 ml of PBS with CaCl_2_ 1% solution was added. Finally, 0.3 g of sodium alginate (Sigma-Aldrich, MO, USA) was added to make a total of 10 ml of hydrogel (3% w/v), which was then incubated for 30 min and then used for printing.

The artificial trachea was constructed with a total of five layers with 20 mm length: first layer, PCL (grid pattern, 5 mm diameter); second layer, hydrogel (cylindrical, 6 mm diameter) with epithelial cells; third layer, PCL (cylindrical, 6.5 mm diameter); fourth layer, hydrogel with chondrocytes (cylindrical, 7 mm diameter); and fifth layer, PCL (grid pattern, 8 mm diameter).

The artificial trachea was imaged using a field emission scanning electron microscope (SEM) with a backscattered electron image detector and an environmental secondary electron detector (JEOL, Ltd., Tokyo, Japan).

### Visualisation of the cell positioning

The position of the printed cells was confirmed using Cell Tracker™ (Life Technologies, OR, USA). Epithelial cells were stained with a green dye (Cell Tracker™ CMFDA, Life Technologies, OR, USA), whereas chondrocytes were stained with a red dye (Cell Tracker™ CMTPX, Life Technologies, OR, USA). Microscopic images were obtained with fluorescence imaging using an Olympus^®^ DP71 microscope digital camera installed on an Olympus® BX51TF system microscope (Olympus, Tokyo, Japan).

### Cell viability test

The artificial trachea was cultured with Dulbecco’s Minimal Essential Medium with high glucose supplemented with 10% FBS, 1% penicillin-streptomycin, 10 µg/ml amphotericin B, and 50 µg/ml gentamicin. Then, cell viability was analysed statistically using a Live/Dead Assay at 1 day, 3 days, 7 days, and 14 days. A total of 16 artificial tracheas were stained with calcein AM (1:500) and ethidium homodimer-1 (1:1000) (Invitrogen) at 37 °C for 1 hour and imaged using confocal microscopy (LSM 700, Zeiss). The number of viable cells and dead cells in each image were quantified using ImageJ software (National Institute of Health, NY, USA). Microscopic images at 400× magnification from each of the eight sites of the samples were used for live cell counting. Live cell ratio (%) was calculated as the ratio of live cell nuclei (green) to the total cell nuclei.

### Anaesthesia and surgical procedure

To evaluate the *in vivo* epithelial and cartilage regeneration efficacy of the artificial trachea, partial resection model was used in New Zealand rabbit. 21 rabbits were used and all rabbits showed normal breathing. As a control group, artificial tracheas without any cells were transplanted into six rabbits. The experimental group was defined as those receiving an artificial trachea with nasal epithelial and auricular cartilage cells. For the experimental group, 6 rabbits were used in the 3-month and 6-month observation groups, and 3 rabbits in the 12-month observation group. Mature male New Zealand White rabbits (3.0–3.5 kg) were injected with 5 mg/kg xylazine and 10 mg/kg Zoletil® intramuscularly at 15 min intervals for anaesthesia. Then, 2% of an isoflurane was used to maintain anaesthesia after inserting the endotracheal tube.

**Figure 7:**
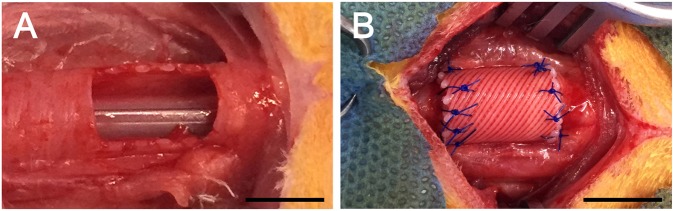
Surgical procedure. (**A**) A picture of the partially resected trachea. The ventral part of the resected trachea is a semi-circular shape. (**B**) The artificial trachea is cut into semi-circular shapes and placed with interrupted sutures. Scale bars indicate 1 cm.

The rabbit was fixed in the dorso-ventral position and then the trachea was approached via a ventral midline incision in the neck. After exposure of the trachea, the ventral portion of the trachea was cut into a semi-cylindrical shape measuring approximately 1.5 × 1.5 cm, and the artificial trachea was cut into a semicircular shape and implanted with 5–0 polyglyconate suture (Maxon™, Covidien, MN, USA) (Fig. 7). Then, the muscular and subcutaneous layers were closed with Maxon 5–0. The skin was closed with 4–0 non-absorbable monofilament sutures.

### Follow-up and evaluation

Radiographs and breathing videos were taken before surgery, immediately after surgery, and every month thereafter for each experimental group. The reduced diameter of the trachea on the radiograph taken just before euthanasia was calculated as a ratio. Breathing videos were 5 min long, and scored for respiratory patterns. The respiration pattern of each group was recorded for 5 min and scored according to the pattern; that is, 0 point for normal breathing, 1 point for intermittent crackles or stridor when excited, 2 points for intermittent crackles or stridor at rest, and 3 points for laboured respiration with continuous crackles or stridor. To observe the diameter of the trachea, radiographs were taken immediately after surgery and every month (VXR - 9 M, DRGRM, Gyeonggi-do, Korea). A computed tomography machine (Brivo 385, GE Health Care, Incheon, Korea) was used to take images just before euthanasia, and images inside the trachea were taken using a bronchoscope (CV 260 S, Olympus, Tokyo, Japan).

### Histologic examination

The artificial trachea obtained by autopsy was cut in the middle and observed internally. Then, they were fixed with 10% phosphate-buffered formalin, embedded in paraffin, sectioned, and stained with haematoxylin and eosin, mason’s trichrome, and safranin O to evaluate the degree of epithelialisation and neocartilage formation.

### Statistical analysis

All data are expressed as mean ± standard deviation. Statistical analyses were performed using GraphPad Prism 5.0 software (GraphPad Software Inc., San Diego, CA, USA). Normal data distribution was determined using the Shapiro-Wilk test. A two-tailed Student’s unpaired t-test was used to compare the mean values of all study parameters. A P value < 0.05 was considered statistically significant.

## Supplementary information

Supplementary Information
