# Supplementary material for: Experimental Tracheal Replacement Using 3-dimensional Bioprinted Artificial Trachea with Autologous Epithelial Cells and Chondrocytes
Source: Sci Rep. 2019 Feb 14;9:2103. doi: 10.1038/s41598-019-38565-z (PMC6375946; doi:10.1038/s41598-019-38565-z)
Supplement: Supplementary file 1 — Supplementary Information [file 41598_2019_38565_MOESM1_ESM.docx]

**Supplementary information**

**Full title:** Experimental Tracheal Replacement Using 3-dimensional Bioprinted Artificial Trachea with Autologous Epithelial Cells and Chondrocytes

**Running headline:** Tracheal replacement with 3-d bioprinted artificial trachea

**Authors:** Jae-Hyun, Park^1, 2^, D.V.M., Jeong-Kee Yoon^3^, Ph.D., Jung Bok, Lee^3^, Ph.D., Young Min Shin^3^, Ph.D., Kang-Woog, Lee^2^, Ph.D., Sang-Woo, Bae^1, 2^, D.V.M., JunHee, Lee^4^, Ph.D., JunJie, Yu^4,5^, Cho-Rok Jung^6^, Ph.D., Young-Nam, Youn^2^, Hwi-Yool, Kim^1^, D.V.M., Ph.D., Dae-Hyun, Kim^2, *^, D.V.M., Ph.D.

**Affiliations:**^1^Department of Veterinary Surgery, College of Veterinary Medicine, Konkuk University, 120 Neungdong-ro, Gwangjin-gu, Seoul 05029, Republic of Korea

^2^Division of Cardiovascular Surgery, Severance Cardiovascular Hospital, Yonsei University College of Medicine, 50-1 Yonsei-ro, Sedaemun-gu, Seoul 03722, Republic of Korea

^3^Severance Biomedical Science Institute, Yonsei University College of Medicine, 50-1 Yonsei-ro, Sedaemun-gu, Seoul 03722, Republic of Korea

^4^Department of Nature-Inspired Nanoconvergence System, Korea Institute of Machinery and Materials, 156 Gajeongbuk-Ro, Yuseong-Gu, Daejeon 34103, Republic of Korea

^5^Department of Biomedical Engineering, School of Integrative Engineering, Chung-Ang University, 84 Heukseok-Ro, Dongjak-Gu, Seoul 06974, Republic of Korea

^6^Gene Therapy Research Unit, Korea Research Institute of Bioscience and Biotechnology, 125 Gwahak-ro, Yuseong-gu, Daejeon, Republic of Korea

***Corresponding Author:** Dae-Hyun, Kim, D.V.M., Ph.D.

Division of Cardiovascular Surgery, Severance Cardiovascular Hospital, Yonsei University College of Medicine, 50-1 Yonsei-ro, Sedaemun-gu, Seoul 03722, Korea

E-mail: vet1982@hanmail.net


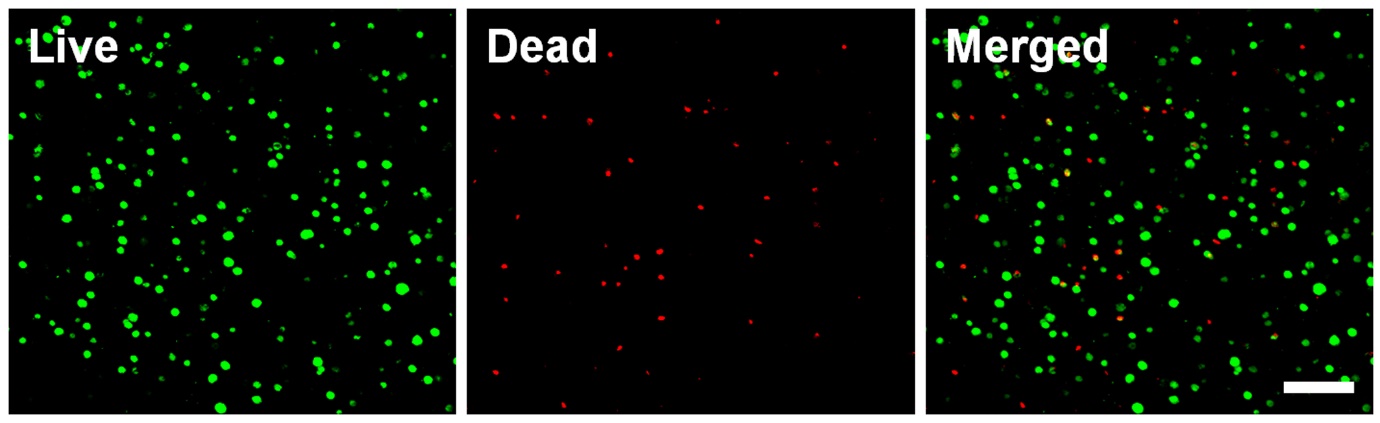


**Supplementary figure S1.** Live and dead assay at 24 hours after cross-linking the sodium alginate hydrogel without 3D-printing. A film-typed thin alginate hydrogel containing cells was formed and the cells were cultured for 24 hours. Live cells and dead cells were stained with Calcein-AM and ethidium homodimer-2, respectively. The percentage of live cell in total at day 1 was 66.02 ± 3.47%, which is similar to that of 3D-printed cells (Fig. 2), indicating that 3D-printing process itself has minimal effect to cell viability. Scale bar indicates 100 μm.
